# CD4^+^ CTLs Act as a Key Effector Population for Allograft Rejection of MSCs in a Donor MHC-II Dependent Manner in Injured Liver

**DOI:** 10.14336/AD.2022.0314

**Published:** 2022-12-01

**Authors:** Shuang Shen, Yuanhui Li, Mengting Jin, Dongdong Fan, Ruolang Pan, Aifu Lin, Ye Chen, Lixin Xiang, Robert Chunhua Zhao, Jianzhong Shao

**Affiliations:** ^1^College of Life Sciences, Key Laboratory for Cell and Gene Engineering of Zhejiang Province, Zhejiang University, Hangzhou, China.; ^2^Institute of Basic Medical Sciences, Chinese Academy of Medical Sciences, School of Basic Medicine, Peking Union Medical College, Beijing, China.; ^3^Division of Medical Genetics and Genomics, the Children's Hospital, Zhejiang University School of Medicine, Hangzhou, China.; ^4^Key Laboratory of Cell-Based Drug and Applied Technology Development in Zhejiang Province, Hangzhou, China.

**Keywords:** mesenchymal stromal/stem cell, major histocompatibility complex class II, CD4^+^ cytotoxic T lymphocyte, allograft rejection, hepatic injury

## Abstract

Mesenchymal stromal/stem cells (MSCs) have been considered an attractive source of cytotherapy due to their promising effects on treating various diseases. Allogeneic MSCs (allo-MSCs) are extensively used in clinical trials due to their convenient preparation and credible performance. Traditionally, allo-MSCs are considered immunoprivileged with minimal immunogenicity and potent immunomodulatory capacity. However, growing evidence has suggested that allo-MSCs also induce immune response and cause rejection after transplantation, but the underlying cellular and molecular mechanisms remain to be elucidated. Here, we demonstrated that allografted MSCs upregulated MHC-II upon stimulation of IFN-γ in hepatic inflammatory environment by using mouse model of CCl_4_-induced liver injury. MHC-II upregulation enhanced the immunogenicity of allo-MSCs, leading to the activation of alloreactive T cells and rejection of allo-MSCs. However, MHC-II deficiency impaired the allogenic reactivity, thereby rescuing the loss of allo-MSCs. Mechanistically, CD4^+^ cytotoxic T lymphocytes (CTLs), rather than CD8^+^ CTLs, acted as the major effector for allo-MSC rejection. Under liver injury condition, the transplanted allo-MSCs upregulated CD80 and PD-L1, and CD8^+^ CTLs highly expressed CTLA-4 and PD-1, thereby inducing immune tolerance of CD8^+^ T cells to allo-MSCs. On the contrary, CD4^+^ CTLs minimally expressed CTLA-4 and PD-1; thus, they remain cytotoxic to allo-MSCs. Consequently, transplantation of MHC-II-deficient allo-MSCs substantially promoted their therapeutic effects in treating liver injury. This study revealed a novel mechanism of MSC allograft rejection mediated by CD4^+^ CTLs in injured liver, which provided new strategies for improving clinical performance of allo-MSCs in benefiting hepatic injury repair.

Mesenchymal stromal/stem cells (MSCs) are multipotent progenitor cells with the potential to differentiate into various cell types of mesodermal origin, including osteocytes, chondrocytes, and adipocytes [1, 2]. MSCs are also recognized to support hematopoiesis, regulate immune responses, and promote tissue regeneration [3-7]. In addition, due to their convenient accessibility and expansion, MSCs are considered attractive candidates for cytotherapy in a series of diseases [8-10]. In the previous decades, MSCs have shown promising effect on treating different diseases, such as graft versus host disease (GVHD), liver cirrhosis and Crohn’s disease [11-13]. The patient-derived MSCs probably have variable performance, and isolation and expansion of MSCs commonly require several weeks; therefore, treatment of autologous MSCs have become limited [14]. On the contrary, allogeneic MSCs (allo-MSCs) from healthy donors can be selected based on potency assays and prepared in advance [15, 16]. As a result, allo-MSCs are widely used in clinical trials [17, 18].

The culture-expanded MSCs express low level of major histocompatibility complex (MHC) class I (MHC-I) and minimal MHC class II (MHC-II) or co-stimulatory molecules (e.g., CD40, CD80 and CD86) [19, 20]. In addition, MSCs demonstrate potent immunomodulatory capacity by modulating various immune cells involved in innate and adaptive immunities [21-24]. These properties enable MSCs to be immunoprivileged for clinical therapeutic application. Nevertheless, growing evidence indicated that allo-MSCs suffer from immune rejection after transplantation [25-27]. For example, allo-MSCs were found to upregulate MHC-I and MHC-II expressions after implantation into infarcted rat myocardia, followed by CD4^+^ T and CD8^+^ T cell infiltration, and eventually led to allograft rejection [28]. CIITA-knockout abolishes MHC-II induction of implanted allo-MSCs, thereby preventing allograft rejection and benefits myocardial repair [29]. These results suggested the enhanced immunogenicity of allo-MSCs after implantation, which is caused by MHC-II upregulation. However, the exact cellular and molecular mechanisms underlying this process remain to be explored.

CD4^+^ cytotoxic T lymphocytes (CD4^+^ CTLs) are a subset of CD4^+^ T cells that express cytolytic molecules, such as Fas ligand (Fas-L), perforin and granzymes upon activation [30-33]. In general, CD4^+^ CTLs are mostly found in patients with chronic virus infections; where CD8^+^ T cells present functional exhaustion [34, 35]. CD4^+^ CTLs also participate in anti-tumor immunity, which requires MHC-II expression on target cells [36, 37]. Considerably, exocytosis of perforin/granzymes is the major pathway of human CD4^+^ CTL-mediated cytotoxicity, whereas murine CD4^+^ CTLs preferentially exploit the Fas/Fas-L pathway [38]. In addition, CD4^+^ T cells can serve as effector cells to mediate cardiac allograft rejection, which requires the donor MHC-II [39]. Moreover, cytolytic effector molecules, such as perforin and/or Fas-L, are necessary for CD4^+^ T cell-mediated allograft rejection [40]. These results indicated the important role of CD4^+^ CTLs in allograft rejection, and the underlying mechanisms should be elucidated. Recent advances have suggested promising therapeutic effects of MSCs in treating liver diseases; this approach is predominantly based on their immunomodulatory properties [41]. However, the retention of implanted MSCs decreases rapidly in liver, thereby restricting their application [42, 43]. Hence, the underlying mechanisms need to be fully characterized. In the present study, we reported the CD4^+^ CTL-mediated immune rejection of allografted MSCs in acutely injured liver by upregulating MHC-II expression. This finding unveiled a novel mechanism of allo-MSC rejection after implantation into injured liver by enhancing immunogenicity through MHC-II upregulation, which provided theoretical basis for improving the clinical potential of allo-MSCs in treating liver injury.

## MATERIALS AND METHODS

### Animals and experimental models

C57BL/6 (H-2^b^), BALB/c (H-2^d^) and BALB/c nude (CByJ.Cg-Foxn1^nu^/J, H-2^d^) mice were obtained from GemPharmatech. C57BL/6 MHC-II^-/-^ (B6.129S2-H2^dlAb1-Ea^/J, H-2^b^) mice were obtained from the Jackson Laboratory. All mice were housed under specific pathogen-free conditions. All the experimental procedures were performed according to institutional guidelines. The 8- to 12-week-old and sex-matched mice were used for the experimental procedures. For acutely liver injury model, BALB/c (H-2^d^) female mice were intraperitoneally injected with CCl_4_ (2.5 ml/kg of body weight; 20% (v/v) in olive oil, Sangon Biotech) every other day for twice. At 24 h after secondary CCl_4_ administration, mice were transplanted with total 1 × 10^6^ MSCs through tail vein or sacrificed for preparation of liver injury-conditioned medium. Experiments were carried out in a blind manner where investigators assessing the outcomes were blinded to the genotype and treatment of the mice. All animal experiments were performed in accordance with legal regulations and approved by a local ethics committee (Ethics Code: ZJU20200110).

### Preparation and culture of MSCs

MSCs were isolated from C57BL/6 (H-2^b^), BALB/c (H-2^d^) and C57BL/6 MHC-II^-/-^ (B6.129S2-H2^dlAb1-Ea^/J, H-2^b^) male mice as previously described [44]. The cells were cultured in Dulbecco’s modified Eagle’s medium (DMEM; Hyclone, cat. no. SH30021.01) supplemented with 10% fetal bovine serum (FBS; Gibco, cat. no. 10091-148) and 1% penicillin/streptomycin (P/S; Gibco, cat. no. 15140-122) and cultured at 37°C in a humidified atmosphere with 5% CO_2_. MSCs were passaged by using 0.25% trypsin-EDTA (Gibco, cat. no. 25200-056) at approximately 90% confluence. MSCs with 3-6 passages were identified by the tri-lineage potential and surface phenotypic markers as previously described [45] and used in experiments.

### Preparation of liver injury-conditioned medium

Acute hepatic injury of BALB/c mice was induced by CCl_4_ as described above. Mice were sacrificed by CO_2_ inhalation, and livers were harvested, cut into pieces with size of approximately 1 mm^3^ and gently washed with PBS. Then the tissue blocks were seeded in a T25 flask (Corning, cat. no. 430639) with a total weight of 500 mg and maintained at 37°C for 2 h in a humidified atmosphere with 5% CO_2_. When tissue blocks adhered to the bottom of the flask, 4 ml DMEM supplemented with 1% FBS and 1% P/S was added in flask, kept covering the tissues and incubated for another 48 h. Supernatant was collected, centrifuged at 5000 g for 10 min and passed through a 0.22-µm filter. The filtrate was finally defined as the liver injury-conditioned medium (LICM) and stored in aliquots at -80 C° for future use.

### MHC-II expression of MSCs induced by LICM and IFN-γ

C57BL/6 (H-2^b^) mice-derived MSCs (2 × 10^5^) were seeded in the 6-well plate one day before induction, then medium was replaced with 10% LICM (diluted with DMEM containing 1% FBS and 1% P/S) and incubated for 1, 2, 4, 7 and 14 days respectively. Alternatively, medium was replaced with 2.5, 5, 10, 15 and 20% LICM respectively and incubated for 2 days. After induction, MSCs were collected for examination of MHC-II expression. To test the role of IFN-γ in LICM-induced MHC-II expression, 10% LICM was pre-treated with 0, 7.5, 15 and 30 ng/ml anti-IFN-γ monoclonal antibody (mAb) (eBioscience, cat. no. 16731185) respectively and incubated at 37°C for 1 h. Non-related rat IgG isotype (30 ng/ml, eBioscience, cat. no. 16430185) was used as control. After seeding, medium was replaced with pre-treated LICM and incubated for 2 days. In addition, IFN-γ-induced MHC-II expression of MSCs was tested by adding different doses (62.5, 125, 250, 500 and 1000 ng/ml) of recombinant murine IFN-γ (Sangon Biotech, cat. no. C600059). After incubation for 2 days, the expression of MHC-II in/on MSCs was examined by quantitative real-time PCR (RT-qPCR), western blot and flow cytometry analysis as previously described [45]. Primers used in RT-qPCR are as follows: MHC-II-Fwd (5’GGCTCAGAAATAGCAAGTCA3’), MHC-II-Rev (5’AATCTCAGGTTCCCAGTGTT3’), GAPDH-Fwd (5’AATGGATTTGGACGCATTGGT3’), GAPDH-Rev (5’TTTGCACTGGTACGTGTTGAT3’). The relative transcriptional expression level of MHC-II gene was calculated using the 2^-ΔΔCt^ method with GAPDH for normalization. Antibodies used in western blot and flow cytometry are as follows: anti-MHC-II polyclonal antibody (Abcam, cat. no. ab180779), anti-GAPDH mAb (Invitrogen, cat. no. 398600), Goat anti-Rabbit IgG HRP polyclonal antibody (Abcam, cat. no. ab6721), Goat anti-mouse IgG HRP secondary antibody (Invitrogen, cat. no. 31430), anti-MHC-II I-A^b^ APC mAb (eBioscience, cat. no. 17532082).

### Expression of co-signaling molecules and Fas of MSCs induced by LICM

C57BL/6 (H-2^b^) mice-derived MSCs were treated without (control) or with 10% LICM for 3 days; then the cells were blocked with 10% rat serum and incubated with anti-CD80 PerCP-eFluor 710 (eBioscience, cat. no. 46080180), anti-CD86 PerCP-eFluor 710 (eBioscience, cat. no. 46086280), anti-PD-L1 PerCP-eFluor 710 (eBioscience, cat. no. 46598280) and anti-Fas APC (eBioscience, cat. no. 17095180) mAbs. Flow cytometry was performed with BD FACSJazz; and data were analyzed with FlowJo software.

### Immunohistochemical staining for MHC-II expression of allografted MSCs

C57BL/6 (H-2^b^) mice-derived MSCs (1 × 10^6^) were labeled with CM-DiI (Invitrogen, cat. no. C7000) and grafted into BALB/c (H-2^d^) mice with CCl_4_-induced acute hepatic injury through tail vein. After transplantation for 1, 3, 7 and 14 days, frozen tissue sections were fixed and incubated with anti-MHC-II I-A^b^ mAb (Santa Cruz, cat. no. sc-32247). The secondary antibody (MultiSciences, cat. no. 05103602) was used according to the manufacturer’s instructions. Nuclei were stained with DAPI. Images were obtained by using fluorescent microscopy (Carl Zeiss Axiostar plus).

### Examination on cellular survival and MHC-II expression of MSCs in vivo

C57BL/6 (H-2^b^) mice-derived MSCs (5 × 10^5^) were labeled with DiO (Biorigin, cat. no. BN14007), and BALB/c (H-2^d^) mice-derived MSCs (5 × 10^5^) were labeled with DiO and DiD (UsEverbright, cat. no. D4019). MSCs with different staining were mixed and grafted into BALB/c (H-2^d^) mice with CCl_4_-induced acute hepatic injury through tail vein. After transplantation for 7 and 14 days, livers were harvested. Hepatic non-parenchymal cells were isolated, blocked with 10% rat serum and then incubated with anti-MHC-II (I-A/I-E) PE mAb (eBioscience, cat. no. 12532181), which recognizes both H-2^b^ and H-2^d^ haplotypes. Non-related rat IgG isotype (eBioscience, cat. no. 12403181) was used as control. Cell numbers and MHC-II expression of MSCs were analyzed by flow cytometry. Likewise, MHC-II^-/-^ C57BL/6 mice-derived MSCs (5 × 10^5^) were labeled with DiO and DiD, wild type C57BL/6 and BALB/c mice-derived MSCs (5 × 10^5^) were labeled with DiO. MSCs with different staining were mixed respectively and grafted into BALB/c (H-2^d^) mice with hepatic injury. Livers were harvested after transplantation for 14 days. Hepatic non-parenchymal cells were isolated and cell numbers of MSCs were analyzed by flow cytometry.

### Isolation of hepatic non-parenchymal cells

Livers were mechanistically disrupted and incubated in the digestive solution (1 × PBS with 100 U/ml DNase I, BioDuly, cat. no. E0046) at 37°C for 20 min. Then, tissues were passed through a sterile 70-μm cell strainer and spun at 50 g for 3 min to remove hepatocytes (pellet). Hepatic non-parenchymal cells in the cell suspension were further enriched by centrifugation at 800 g for 30 min in a 2-step Percoll gradient (20% and 80%). Finally, hepatic non-parenchymal cells were harvested and resuspended in PBS for subsequent experiments.

### Proliferation and cytotoxicity of leukocytes

Before co-culture, wild type (WT) and MHC-II^-/-^ C57BL/6 mice-derived MSCs were treated without (control) or with 10% LICM for 3 days. For proliferation assay, MSCs (4 × 10^4^) were seeded in the 12-well plate. Splenic leukocytes (4 × 10^5^) were isolated from BALB/c mice, labeled with CFSE and co-cultured with untreated WT MSCs or LICM-treated WT MSCs or LICM-treated MHC-II^-/-^ MSCs, respectively. After 3 days, flow cytometry was performed. For cytotoxicity assay, MSCs (5 × 10^3^) were seeded in the 96-well plate. Splenic leukocytes (5 × 10^4^) were isolated from BALB/c mice and co-cultured with untreated WT MSCs or LICM-treated WT MSCs or LICM-treated MHC-II^-/-^ MSCs respectively for 3 days. The cytotoxicity was evaluated by measuring LDH release with a commercial kit (Progema, cat. no. G1780) according to the manufacture’s instruction.

### Proliferation and cytotoxicity of CD4^+^ CTLs

For proliferation assay, MSCs (4 × 10^4^) were seeded in the 12-well plate. Splenic leukocytes (4 × 10^5^) were isolated from BALB/c mice and co-cultured with untreated WT MSCs or LICM-treated WT MSCs or LICM-treated MHC-II^-/-^ MSCs respectively for 5 days. Then, the cells were blocked with 10% rat serum and incubated with anti-CD45 FITC (eBioscience, cat. no. 11045182), anti-CD4 PE (eBioscience, cat. no. 12004182) and anti-Fas-L APC (eBioscience, cat. no. 17591180) mAbs. The propotion of CD4^+^Fas-L^+^ CTLs was examined by flow cytometry. For detection of CD4^+^Perforin^+^ CTLs, leukocytes were treated with Brefeldin A (eBioscience, cat. no. 00450651) for the last 5 hours of co-culture. Then, the cells were blocked with 10% rat serum and incubated with anti-CD45 FITC and anti-CD4 PE mAbs. After fixation with 1% paraformaldehyde at room temperature for 10min, leukocytes were treated with 0.1% saponin (Sigma, cat. no. SAE0073) for permeablization at room temperature for 10 min. Next, leukocytes were resuspended in PBS (containing 0.03% saponin and 3% rat serum) and incubated with anti-Perforin APC mAb (eBioscience, cat. no. 17939280) at 4°C for 30 min. The propotion of CD4^+^Perforin^+^ CTLs was analyzed by flow cytometry. For cytotoxicity assay, C57BL/6 mice-derived MSCs (1 × 10^6^) were subcutaneously injected in BALB/c mice. After immunization for 7 days, leukocytes were isolated from spleen, blocked with 10% rat serum and incubated with anti-CD45 FITC, anti-CD4 PE and anti-Fas-L APC mAb. CD4^+^Fas-L^+^ CTLs were sorted by FACS. MSCs (5 × 10^3^) were pre-seeded in the 96-well plate, and then CD4^+^Fas-L^+^ CTLs (2 × 10^4^) were co-cultured with untreated WT MSCs or LICM-treated WT MSCs or LICM-treated MHC-II^-/-^ MSCs respectively for 12 h. The cytotoxicity was evaluated by measuring LDH release as above described.

### Infiltration of immune cells and survival of allo-MSCs in Matrigel

Before transplantation, untreated WT MSCs, LICM-treated WT MSCs and LICM-treated MHC-II^-/-^ MSCs were suspended in Matrigel (Corning, cat. no. 356231) respectively. The MSCs-Matrigel suspension (5 × 10^5^ MSCs in 500 μl Matrigel) was subcutaneously injected in allogeneic BALB/c mice. After 7 days, Matrigels were isolated, mechanistically disrupted and incubated with digestive solution containing 1.6 mg/ml collagenase IV (Sigma, cat. no. C5138) and 100 U/ml DNase I at 37°C for 2 h. Debris was removed by centrifugation at 500 g for 15 min in 30% Percoll and cells (pellet) were collected. To detect CD4^+^ CTL infiltration, cells were treated with Brefeldin A for 5 h, blocked with 10% rat serum and incubated with anti-Fas-L FITC (eBioscience, cat. no. 11591180) and anti-CD4 PE mAbs. After fixation and permeablization, cells were incubated with anti-Perforin APC mAb as described above. Cell numbers of CD4^+^Fas-L^+^ CTLs and CD4^+^Perforin^+^ CTLs in Matrigel was analyzed by flow cytometry. To detect activated CD8^+^ T cell infiltration, cells were blocked with 10% rat serum and incubated with anti-CD45 FITC, anti-CD8 PerCP-Cyanine5.5 (eBioscience, cat. no. 45008180) and anti-CD107a eFluor 660 (eBioscience, cat. no. 50107182) mAbs. Cell number of CD8^+^CD107a^+^ T cells in Matrigel was analyzed by flow cytometry. To detect activated B cell infiltration, cells were blocked with 10% rat serum and incubated with anti-CD45 FITC, anti-CD19 APC (eBioscience, cat. no. 17019382) and anti-CD86 PerCP-eFluor 710 mAbs. Cell number of CD19^+^CD86^+^ B cells in Matrigel was analyzed by flow cytometry. To detect allo-MSC survival, cells were blocked with 10% rat serum and incubated with anti-MHC-I (H-2K^b^) PE mAb (eBioscience, cat. no. 12595882). Cell number of allo-MSCs in Matrigel was analyzed by flow cytometry.

**Figure 1.**
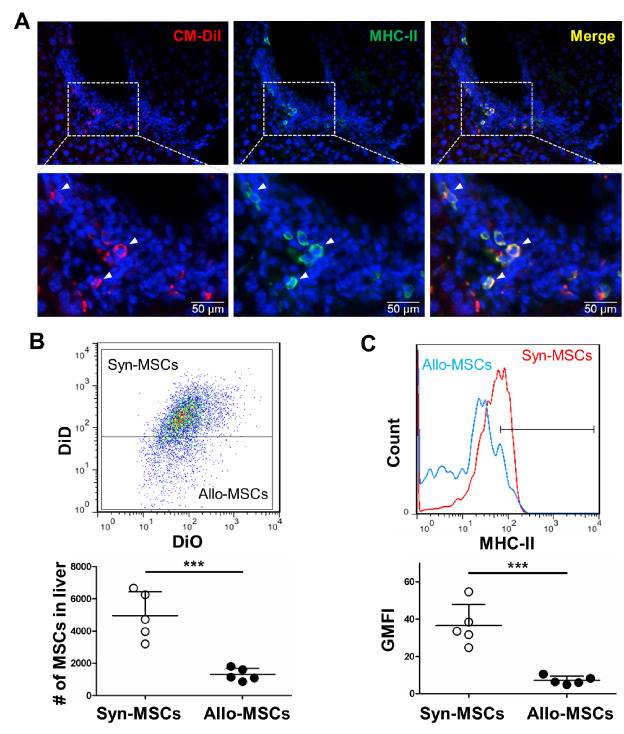
Allografted MSCs upregulate MHC-II expression in acutely injured liver. (A) immunostaining showed MHC-II (green) upregulation of allo-MSCs (red) after transplantation for 14 days in CCl_4_-induced acutely injured liver (arrows indicate MHC-II^+^ allo-MSCs). (B) flow cytometry detected cell numbers of surviving syn-MSCs (DiO^+^DiD^+^) and allo-MSCs (DiO^+^DiD^-^) in acutely injured liver after transplantation for 14 days (n = 5). The results are representative of at least three independent experiments as mean ± SD. ^***^p < 0.001. (C) flow cytometry detected MHC-II expression of surviving syn-MSCs (DiO^+^DiD^+^) and allo-MSCs (DiO^+^DiD^-^) in acutely injured liver after transplantation for 14 days (n = 5). The results are representative of at least three independent experiments as mean ± SD. ^***^p < 0.001.

**Figure 2.**
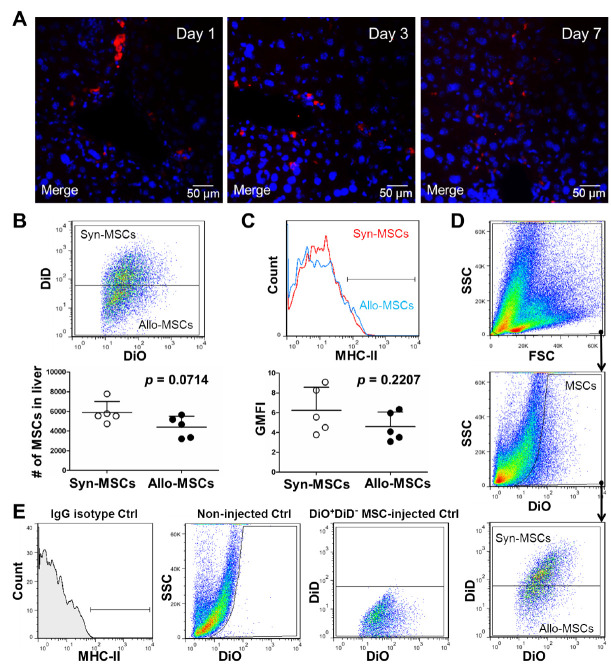
No significant expression of MHC-II on allo-MSCs at early time points after transplantation. (A) immunostaining showed no significant expression of MHC-II (green) was detected on allo-MSCs (red) at early time points (day 1, 3 and 7) after transplantation. (B) flow cytometry detected cell numbers of surviving syn-MSCs (DiO^+^DiD^+^) and allo-MSCs (DiO^+^DiD^-^) in acutely injured liver after transplantation for 7 days (n = 5). The results are representative of at least three independent experiments as mean ± SD. *p*=0.0714. (C) flow cytometry detected MHC-II expression of survived syn-MSCs (DiO^+^DiD^+^) and allo-MSCs (DiO^+^DiD^-^) in acutely injured liver after transplantation for 7 days (n = 5). The results are representative of at least three independent experiments as mean ± SD. *p*=0.2207. (D and E) gating strategy of flow cytometric analysis for transplanted MSCs in acutely injured liver.

### Infiltration of immune cells and survival of allo-MSCs in Matrigel implanted in nude mice

Wild type (WT) and MHC-II^-/-^ C57BL/6 mice-derived MSCs were treated with 10% LICM for 3 days. After induction, WT and MHC-II^-/-^ MSCs (5 × 10^5^) were suspended in Matrigel (500 μl) and subcutaneously injected in BALB/c nude mice respectively. Then, recipient mice were adoptively transferred without (control) or with CD45^+^CD4^-^ leukocytes (CD45^+^ leukocytes with removal of CD4^+^ T subset by FACS) or CD4^+^Fas-L^+^ CTLs by intraperitoneal injection one day after allo-MSC administration. The CD45^+^CD4^-^ leukocytes and CD4^+^Fas-L^+^ CTLs were sorted from immunized BALB/c mice as described above. After engraftment of allo-MSCs for 7 days, cells in Matrigel were isolated; the numbers of CD8^+^CD107a^+^ T cells, CD19^+^CD86^+^ B cells and allo-MSCs were analyzed by flow cytometry.

### Expression of co-inhibitory receptors and survival of CD4^+^/CD8^+^ CTLs

C57BL/6 mice-derived MSCs (1 × 10^6^) were treated with LICM and subcutaneously injected in BALB/c mice for immunization. After 7 days, leukocytes were isolated from spleen. The proportion of CD4^+^Fas-L^+^ and CD4^+^Perforin^+^ CTLs was identified with anti-Fas-L APC, anti-CD4 FITC (eBioscience, cat. no. 11004181) and anti-Perforin APC mAbs as described above. Likewise, the proportion of CD8^+^GzmB^+^ and CD8^+^Perforin^+^ CTLs was identified with anti-GzmB APC (eBioscience, cat. no. 17889880), anti-CD8 FITC (eBioscience, cat. no. 11008181) and anti-Perforin APC mAbs. Expression of CTLA-4 and PD-1 on CD4^+^/CD8^+^ CLTs was analyzed with anti-CTLA-4 PE (eBioscience, cat. no. 12152281) and anti-PD-1 PerCP-eFluor 710 (eBioscience, cat. no. 46998580) mAbs by flow cytometry. For survival analysis, splenic leukocytes were isolated after immunization. LICM-treated allo-MSCs (2 × 10^5^) were co-cultured with leukocytes (1 × 10^6^) in 6-well plate. After 3 days, the proportion of CD4^+^ and CD8^+^ CTLs was identified with anti-CD4 PE, anti-CD8 PE (eBioscience, cat. no. 12008181), anti-Fas-L APC, anti-Perforin APC and anti-GzmB APC mAbs as described above. Cell survival was detected with Annexin V-FITC/7-AAD kit (Beijing BioRab Technology, cat. no. HR8280) by flow cytometry.

### Biochemical and histological examination for liver function

WT and MHC-II^-/-^ C57BL/6 mice-derived MSCs were isolated and expended. BALB/c mice were intraperitoneally injected with 20% CCl_4_ (2.5 ml/kg of body weight) every other day for twice. At 24 h after secondary CCl_4_ administration, MSCs (1 × 10^6^) were transplanted into BALB/c mice with acute hepatic injury via the tail vein. Recipient mice were treated with a third and fourth CCl_4_ injection at day 5 and 10 after MSC transplantation. Meanwhile, non-transplantation and healthy mice were set up as controls. On the 14^th^ day after MSC transplantation, mice were sacrificed following a 12-h fast. Blood serum was obtained, and alanine aminotransferase (ALT) level was measured by using commercially available kit (Nanjing Jiancheng Bioengineering Institutecat, cat. no. C009-2-1). Liver tissue was fixed and embedded in paraffin, sectioned into 5 μm slices and stained with hematoxylin and eosin (H&E). Bright-field images were captured and analyzed by ImageJ software.

### Statistical analysis

The data in this study were presented as the mean ± SD of each group. Statistical analysis was performed using GraphPad Prism 5 software. Statistical significance between two groups was assessed by two-tailed t test (passed normality test) or the non-parametric Mann-Whitney U test (did not passed normality test or n < 6). A value of p < 0.05 was considered statistically significant. The samples were randomly assigned to experimental groups. The investigator was blinded to the group allocation during the experiment and when assessing the outcome as far as possible. All experiments were replicated at least three times.

## RESULTS

### Upregulation of MHC-II on allografted MSCs in injured liver

To investigate the induced expression of MHC-II on allografted MSCs in injured mouse liver, C57BL/6 (H-2^b^) mice-derived MSCs, which hardly express MHC-II before transplantation, were labeled with CM-DiI and intravenously transplanted into BALB/c (H-2^d^) recipient mice with CCl_4_-induced acute hepatic injury (Supplementary Fig. 1A). Immunohistochemical staining was performed with an antibody specific to donor cells (MHC-II H-2^b^). The allografted MSCs exhibited an upregulated expression of MHC-II after transplantation for 14 days (Fig. 1A). Instead, minimal expression of MHC-II was detected at early time points (Fig. 2A). To further determine the correlation between MHC-II expression and MSC allograft rejection, liver-injured BALB/c mice were administered intravenously with a 1:1 mixture of DiO and DiD-labeled BALB/c mice-derived MSCs (DiO^+^DiD^+^ syn-MSCs) and DiO-labeled C57BL/6 mice-derived MSCs (DiO^+^DiD^-^ allo-MSCs). After transplantation for 7 and 14 days, hepatic non-parenchymal cells were collected, and labeled with anti-MHC-II antibody (recognizes both H-2^d^ and H-2^b^ haplotypes). Cell numbers and MHC-II expression levels of syn-MSCs and allo-MSCs were determined by flow cytometry. Result showed significant decrease in the survival of allo-MSCs compared with syn-MSCs at day 14 after transplantation (Fig. 1B); while minimal decrease in cell survival was detected at day 7 after transplantation (Fig. 2B). Interestingly, survived allo-MSCs showed significant low level of MHC-II expression compared with syn-MSCs at day 14 after transplantation (Fig. 1C), whereas no significance of MHC-II expression was detected at day 7 after transplantation (Fig. 2C). As a negative control, minimal MSCs were implanted into the non-injured liver, which is consistent with the previous observation that recruitment of MSCs requires release of chemokines from injured injury (Supplementary Fig. 1B) [46]. These results indicated the recruitment and MHC-II upregulation of allografted MSCs in acutely injured liver and implied the important role of MHC-II upregulation in MSC allograft rejection.

**Figure 3.**
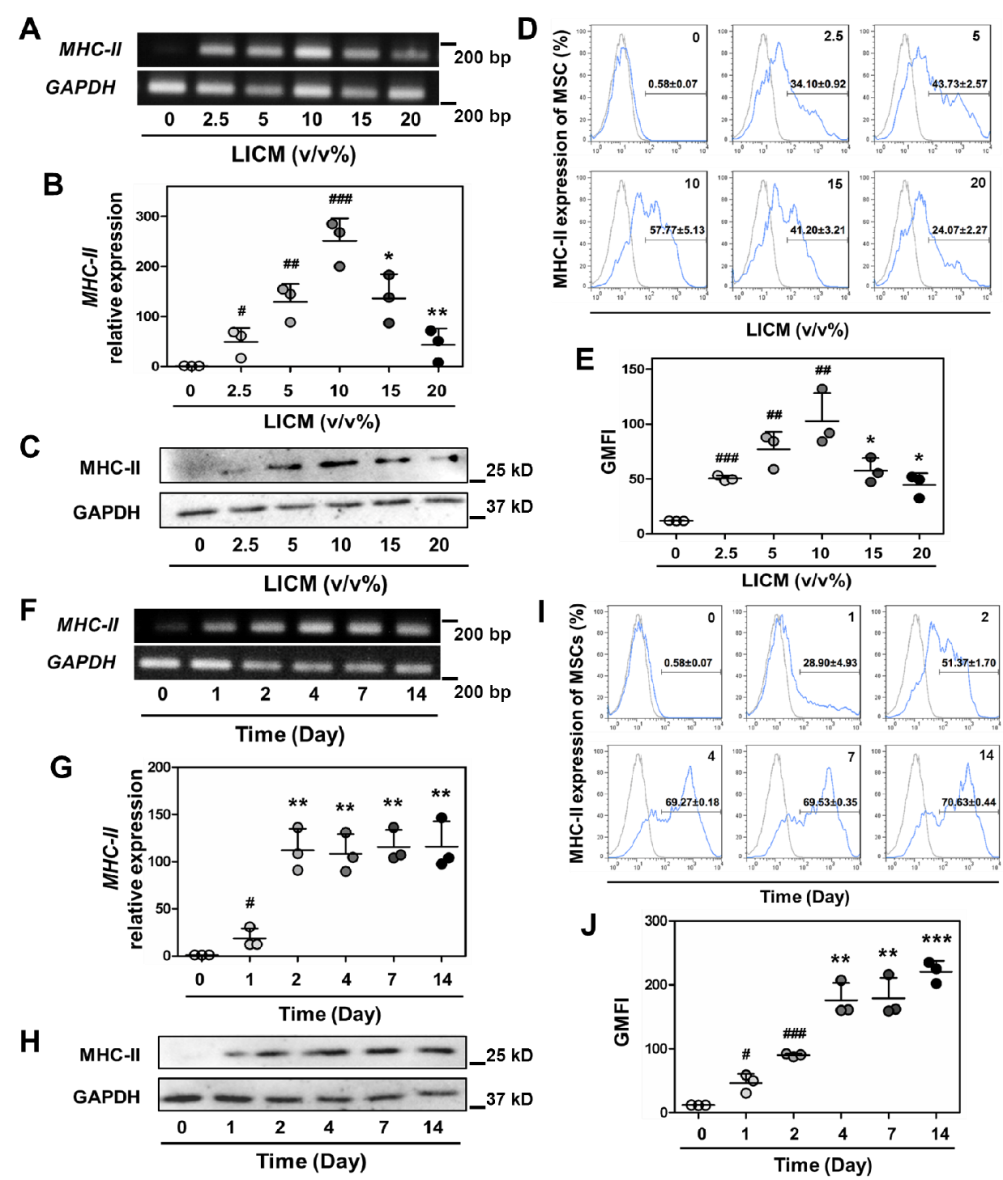
Examination on LICM induced MHC-II expression of MSCs. (A and B) real-time PCR detected MHC-II expression of MSCs after treatment of LICM with different concentrations (n = 3). The results are representative of three independent experiments as mean ± SD. ^#^*p* < 0.05, ^##^*p* < 0.01,^###^*p* < 0.001 *vs.* 0% LICM; ^*^p < 0.05, ^**^p < 0.01 *vs.* 10% LICM. (C) western blot analysis showed MHC-II expression of MSCs after treatment of LICM with different concentrations. (D and E) flow cytometry was performed to analyze MHC-II expression on MSCs after treatment of LICM with different concentrations (n = 3). The results are representative of three independent experiments as mean ± SD. ^##^*p* < 0.01,^###^*p* < 0.001 *vs.* 0% LICM; ^*^p < 0.05 *vs.* 10% LICM. *F* and (G) real-time PCR detected MHC-II expression of MSCs at different time points after LICM induction (n = 3). The results are representative of three independent experiments as mean ± SD. ^#^*p* < 0.05 *vs.* Day 0; ^**^p < 0.01 *vs.* Day 1. (H) western blot analysis showed MHC-II expression of MSCs at different time points after LICM induction. (I and J) flow cytometry was performed to analyze MHC-II expression on MSCs at different time points after LICM induction (n = 3). The results are representative of three independent experiments as mean ± SD. ^#^*p* < 0.05, ^###^*p* < 0.001 *vs.* Day 0; ^**^p < 0.01, ^***^p < 0.001 *vs.* Day 2.

**Figure 4.**
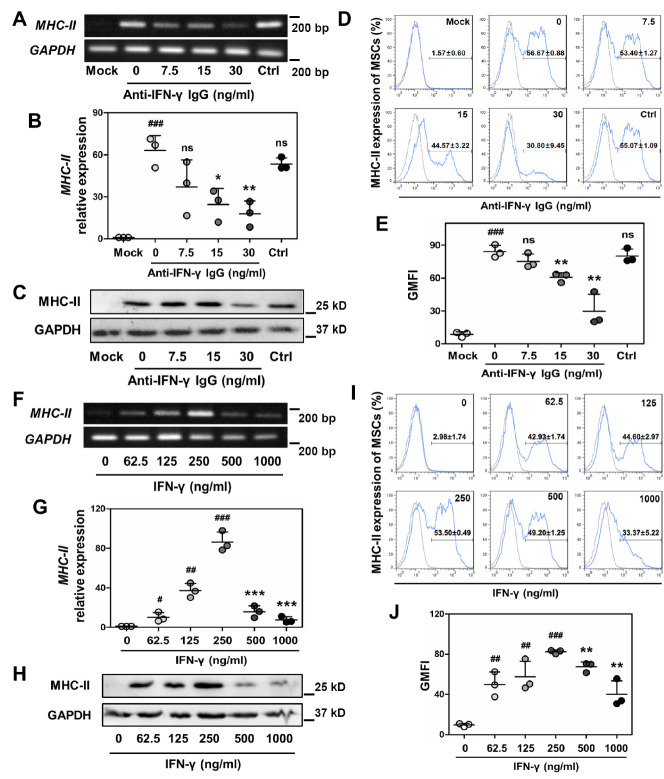
Identification of the regulatory role of IFN-γ in MHC-II expression of MSCs. (A and B) LICM was pre-treated with different doses of anti-IFN-γ monoclonal antibody (mAb); then LICM-induced MHC-II expression of MSCs was detected by real-time PCR (n = 3). Mock, MSCs without LICM induction. Ctrl, LICM pre-treated with rat IgG (isotype control). The results are representative of three independent experiments as mean ± SD. ^###^*p* < 0.001 *vs.* Mock; ns = non-significant, ^*^p < 0.05, ^**^p < 0.01 *vs.* 0 ng/ml anti-IFN-γ IgG. (C-E) western blot (C) and flow cytometry (D and E) were performed to analyze MHC-II expression of MSCs after induction of LICM with different doses of anti-IFN-γ mAb pre-treatment (n = 3). The results are representative of three independent experiments as mean ± SD.^###^*p* < 0.001 *vs.* Mock; ns = non-significant, ^**^p < 0.01 *vs.* 0 ng/ml anti-IFN-γ IgG. (F and G) real-time PCR detected MHC-II expression of MSCs after treatment of IFN-γ with different concentrations (n = 3). The results are representative of three independent experiments as mean ± SD. ^#^*p* < 0.05, ^##^*p* < 0.01,^###^*p* < 0.001 *vs.* 0 ng/ml IFN-γ; ^***^p < 0.001 *vs.* 250 ng/ml IFN-γ. (H-J) western blot (H) and flow cytometry (I and J) were performed to analyze MHC-II expression of MSCs after treatment of IFN-γ with different concentrations (n = 3). The results are representative of three independent experiments as mean ± SD. ^##^*p* < 0.01,^###^*p* < 0.001 *vs.* 0 ng/ml IFN-γ; ^**^p < 0.01 *vs.* 250 ng/ml IFN-γ.

### Role of IFN-γ in MHC-II induction of allografted MSCs under liver injury condition

To identify the key factor that upregulates MHC-II expression of allografted MSCs in injured liver, we initially prepared liver injury-conditioned medium (LICM) that simulated the acute liver injury condition *in vitro*. RT-qPCR and Western blotting analyses showed that MSCs exhibited a remarkably increased MHC-II expression at both mRNA and protein levels after treatment of LICM with concentrations ranging from 2.5% to 10%, while a decreased MHC-II expression with concentrations of LICM from 15% to 20% (Fig. 3A-C and Supplementary Fig. 2). Flow cytometric analysis demonstrated the same changes in MHC-II expression on the cell membrane (Fig. 3D and E). These results suggested that MHC-II was upregulated in a dose-dependent manner within a narrow window at low levels of LICM, while downregulated after treatment with high concentrations of LICM. Furthermore, MHC-II was upregulated with the time lasting of LICM treatment with an optimized concentration (10%) and reached the maximum on day 2 at mRNA level and day 4 at protein level (Fig. 3F-J). This observation suggested that MHC-II can be rapidly induced during a two/four-day period under an appropriate concentration of LICM. Previous studies indicated a robustly increased inflammatory cytokines in injured liver induced by CCl_4_, mainly including IL-6, TNF-α and IFN-γ [47]. In addition, the effect that IFN-γ upregulates MHC-II expression of MSCs had been illustrated by *in vitro* experiments [48, 49]. Thus, we hypothesized that IFN-γ may play an important role in LICM-induced MHC-II upregulation of MSCs.

**Figure 5.**
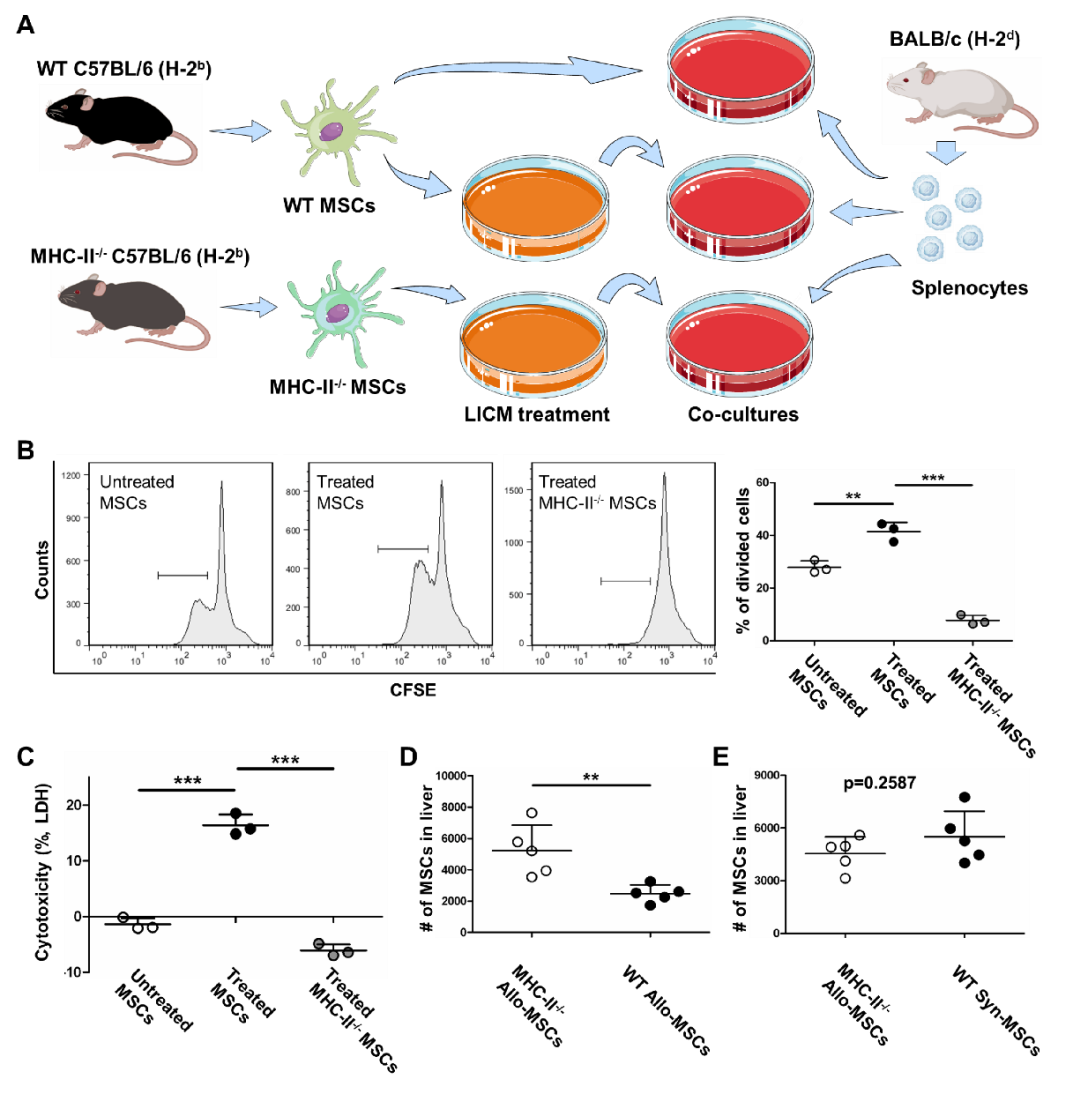
Allo-MSCs with MHC-II upregulation activated proliferation and cytotoxicity of splenocytes and suffered graft-rejection. (A) schematic for co-cultures of splenocytes and allo-MSCs pre-treated with/without LICM. (B) splenocytes were labeled with CFSE and co-cultured with untreated MSCs, LICM-treated MSCs and LICM-treated MHC-II^-/-^ MSCs respectively; flow cytometry was performed to analyze proliferation of splenocytes (n = 3). The results are representative of three independent experiments as mean ± SD. ^**^p < 0.01, ^***^p < 0.001. (C) splenocytes were co-cultured with untreated MSCs, LICM-treated MSCs and LICM-treated MHC-II^-/-^ MSCs respectively; cytotoxicity of splenocytes was determined by LDH release (n = 3). The results are representative of three independent experiments as mean ± SD. ^***^p < 0.001. (D) flow cytometry detected cell numbers of survived MHC-II^-/-^ allo-MSCs and WT allo-MSCs in injured liver after transplantation for 14 days (n = 5). The results are representative of at least three independent experiments as mean ± SD. ^**^p < 0.01. (E) flow cytometry detected cell numbers of survived MHC-II^-/-^ allo-MSCs and WT syn-MSCs in injured liver after transplantation for 14 days (n = 5). The results are representative of at least three independent experiments as mean ± SD. *p*=0.2587.

To confirm this notion, IFN-γ was blocked by adding anti-IFN-γ antibody in LICM prior to treating MSCs. As expected, blocking of IFN-γ resulted in decreased MHC-II induction of MSCs at mRNA and protein levels by LICM treatment, whereas isotype antibody control did not appear to exert any inhibitory effect (Fig. 4A-C). Consistent results were obtained by flow cytometric analysis (Fig. 4D and E). To further confirm the functional role of IFN-γ in upregulating MHC-II expression, MSCs were treated with different concentrations of recombinant IFN-γ protein. Results showed that MHC-II expression was significantly increased with low levels of IFN-γ (62.5-250 ng/ml) treatment, while there was a dramatic decrease in MHC-II expression accompanied by elevated levels of IFN-γ (500-1000 ng/ml) (Fig. 4F-J). These findings were consistent with the pattern of LICM-induced MHC-II expression, which further defined the critical role of IFN-γ in LICM-induced MHC-II upregulation of MSCs.

### MHC-II upregulation enhanced the immunogenicity and allograft rejection of MSCs

To determine whether MHC-II upregulation of allo-MSCs leads to immune rejection, leukocyte proliferation and cytotoxicity were measured in co-cultures of splenocytes with allo-MSCs (Fig. 5A). After LICM treatment, MSCs caused a remarkably increased proliferation of splenocytes, whereas LICM-treated MHC-II^-/-^ MSCs almost did not lead to splenocyte proliferation (Fig. 5B). Accordingly, LICM-treated MSCs provoked a significant cytotoxicity of splenocytes in contrast with untreated MSCs or LICM-treated MHC-II^-/-^ MSCs (Fig. 5C). To evaluate immune rejection in vivo, liver-injured BALB/c mice received intravenous transplantation with MHC-II^-/-^ C57BL/6 mice-derived MSCs (MHC-II^-/-^ allo-MSCs) and wild-type (WT) C57BL/6 mice-derived MSCs (WT allo-MSCs). After transplantation for 14 days, hepatic non-parenchymal cells were collected; cell numbers of MHC-II^-/-^ allo-MSCs and WT allo-MSCs were determined by flow cytometry. The result showed a significantly increased survival of MHC-II^-/-^ allo-MSCs compared with WT allo-MSCs (Fig. 5D). Also, no significant difference in cell numbers was detected between MHC-II^-/-^ allo-MSCs and WT syn-MSCs (Fig. 5E). These results demonstrated that MHC-II upregulation enhanced the immunogenicity of allografted MSCs, and finally resulted in immune rejection.

### CD4^+^ CTLs mediated MSC allograft rejection in a donor MHC-II dependent manner

**Figure 6.**
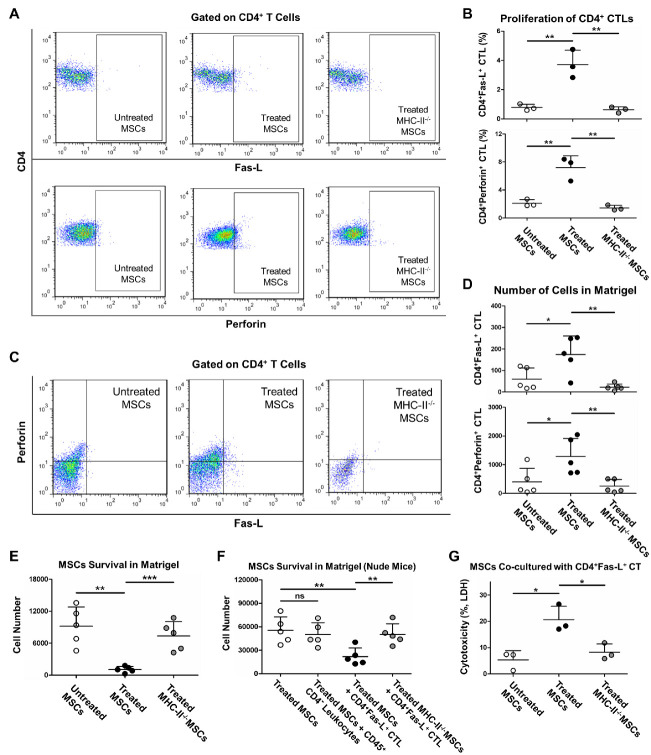
CD4^+^ CTLs mediated allograft rejection of MSCs with MHC-II upregulation. (A and B) after co-culture with untreated MSCs, LICM-treated MSCs and LICM-treated MHC-II^-/-^ MSCs respectively, allogeneic splenocytes were labeled with fluorochrome-conjugated anti-CD45, CD4, Fas-L, Perforin mAbs, then proliferation of CD4^+^ CTLs (ratio of CD4^+^Fas-L^+^ T cell and CD4^+^Perforin^+^ T cell in total CD4^+^ T cells) was analyzed by flow cytometry (n = 3). The results are representative of three independent experiments as mean ± SD. ^**^p < 0.01. (C and D) untreated MSCs, LICM-treated MSCs and LICM-treated MHC-II^-/-^ MSCs were mixed with Matrigel respectively and injected subcutaneously in allogeneic mice; Matrigel was retrieved 7 days after injection, infiltrated cells were isolated and labeled with anti-CD4, Fas-L, Perforin mAbs, cell numbers of infiltrated CD4^+^ CTLs (CD4^+^Fas-L^+^ T cell and CD4^+^Perforin^+^ T cell) were analyzed by flow cytometry (n = 5). The results are representative of at least three independent experiments as mean ± SD. ^*^p < 0.05, ^**^p < 0.01. (E) MSCs survival in Matrigel was determined after injection, cells in retrieved Matrigel were isolated and labeled with anti-MHC-I mAb (specific to H-2^b^ haplotype), cell numbers of survived MSCs were analyzed by flow cytometry (n = 5). The results are representative of at least three independent experiments as mean ± SD. ^**^p < 0.01, ^***^p < 0.001. (F) MSCs and MHC-II^-/-^ MSCs were treated with LICM, mixed with Matrigel and injected subcutaneously in allogeneic nude mice with/without adoptive transfer of CD45^+^CD4^-^ leukocytes (CD45^+^ leukocytes with removal of CD4^+^ T cells) or CD4^+^Fas-L^+^ CTL. Cell numbers of survived MSCs in Matrigel were analyzed by flow cytometry (n = 5). The results are representative of at least three independent experiments as mean ± SD. ^**^p < 0.01, ns = non-significant. (G) MSCs were treated with LICM, injected subcutaneously in allogeneic mice for 7 days (immunization). Splenocytes were isolated and labeled with anti-CD4, Fas-L mAbs, CD4^+^Fas-L^+^ CTL was sorted by FACS and co-cultured with LICM-treated MSCs, cytotoxicity of CD4^+^Fas-L^+^ CTL was determined by LDH release (n = 3). The results are representative of three independent experiments as mean ± SD. ^*^p < 0.05.

Previous studies revealed the important role of CD4^+^ CTLs in allograft rejection, which required donor MHC-II expression [39, 40]. Here we measured the proliferation of CD4^+^ CTLs in co-cultures of splenocytes with allo-MSCs to evaluate the potential function of CD4^+^ CTLs in MSC allograft rejection. After LICM treatment, MSCs caused a remarkably increased proliferation of CD4^+^Fas-L^+^ CTL and CD4^+^Perforin^+^ CTL. However, the increase in proliferation was impaired with MHC-II knockout (Fig. 6A and B). The infiltration of CD4^+^ CTLs was then measured in vivo. BALB/c mice were implanted subcutaneously with C57BL/6 mice-derived MSCs, which were premixed in Matrigel. In parallel, recipient BALB/c mice likewise received implants of LICM-treated MSCs or LICM-treated MHC-II^-/-^ MSCs, respectively. Results showed that LICM-treated MSCs attracted much more recruitment of CD4^+^Fas-L^+^ CTL and CD4^+^Perforin^+^ CTL than untreated MSCs, whereas MHC-II knockout resulted in remarkably less infiltration of CD4^+^Fas-L^+^ CTL and CD4^+^Perforin^+^ CTL (Fig. 6, C and D). Meanwhile, the cell number of survived MSCs in Matrigel was determined by flow cytometry. As expected, LICM-treated MSCs showed significantly decreased survival compared with untreated MSCs, whereas MHC-II knockout rescued the loss of MSCs greatly in Matrigel (Fig. 6E). To explore whether other immune cells were recruited into the Matrigel, which may potentially involve in allo-MSC rejection, we examined the infiltration of activated CD8^+^ T cells and B cells in Matrigel, two major downstream target subsets for CD4^+^ T cells in allo-immunity. Consequently, no significant infiltration of CD8^+^CD107a^+^ T cells and CD19^+^CD86^+^ B cells was detected after engraftment of MSCs or MHC-II^-/-^ MSCs with/without LICM treatment (Supplementary Fig. 3). These results suggested the major role of CD4^+^ CTLs in MSC allograft rejection. For further clarification, BALB/c nude mice received MSCs which were premixed in Matrigel, with/without adoptive transfer of CD4^+^Fas-L^+^ CTLs or CD45^+^CD4^-^ leukocytes, the latter of which containing various immune cells but with removal of CD4^+^ T cells by FACS. The cell number of survived MSCs in Matrigel was determined by flow cytometry. Consequently, adoptive transfer of CD45^+^CD4^-^ leukocytes showed no significant decline in LICM-treated MSC survival; by contrast, adoptive transfer of CD4^+^Fas-L^+^ CTLs led to a significant decrease in LICM-treated MSC survival, whereas MHC-II knockout dramatically rescued the loss of MSCs (Fig. 6F). In addition, adoptive transfer of CD45^+^CD4^-^ leukocytes did not lead to remarkable infiltration of CD8^+^CD107a^+^ T cells and CD19^+^CD86^+^ B cells in Matrigel implanted in nude mice (Supplementary Fig. 4). Furthermore, CD4^+^ CTL-mediated cytotoxicity was measured in co-cultures of allo-MSCs with sorted CD4^+^Fas-L^+^ CTLs. As expected, LICM-treated MSCs provoked a significantly increased cytotoxicity of CD4^+^Fas-L^+^ CTLs contrary to untreated MSCs or LICM-treated MHC-II^-/-^ MSCs (Fig. 6G). As a result, our study suggested CD4^+^ CTLs as the key effector cells to mediate MSC allograft rejection, which requires the donor MHC-II.

### Involvement of CD4^+^ CTLs rather than CD8^+^ CTLs in MSC allograft rejection

**Figure 7.**
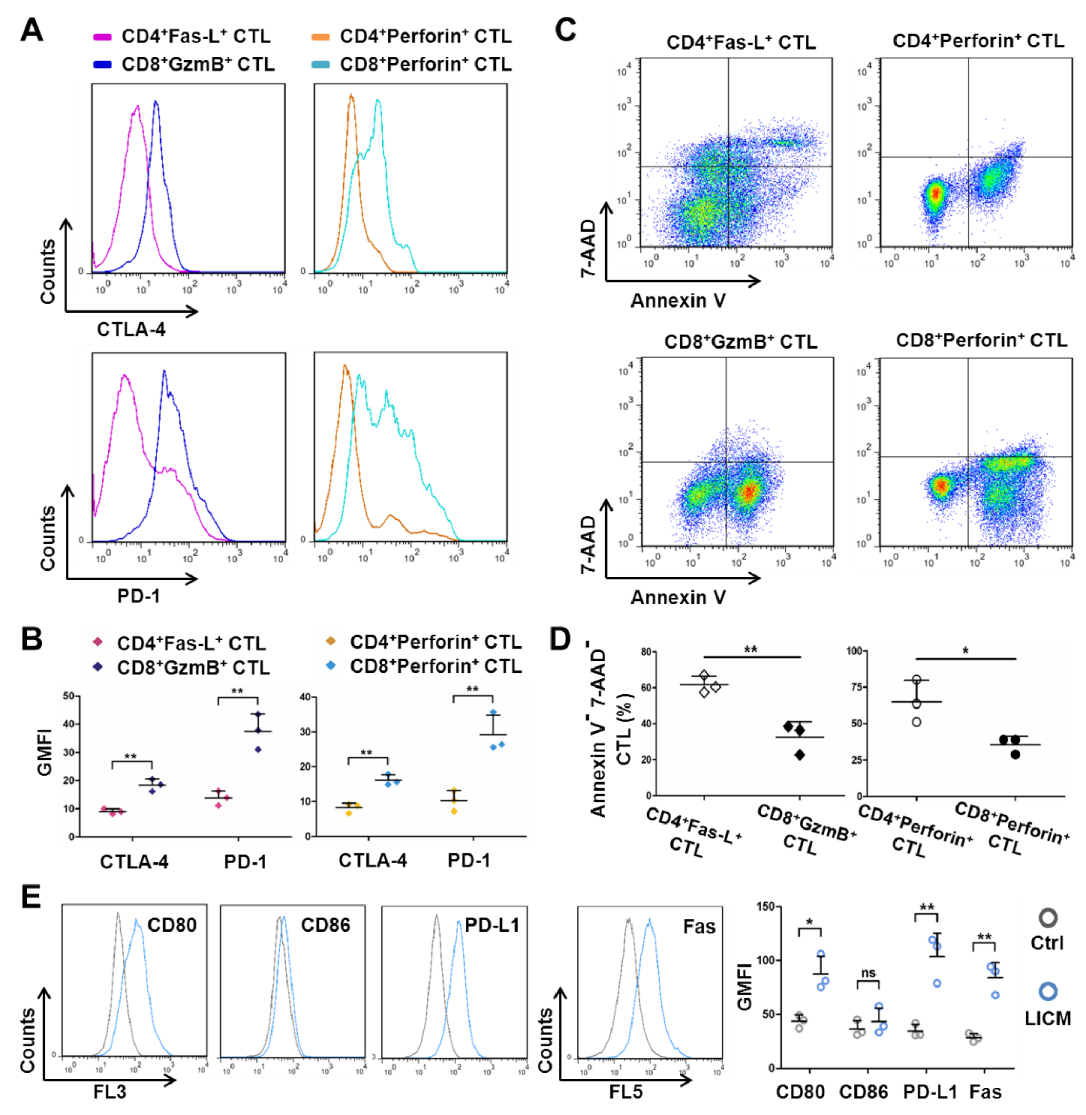
MSCs induced dysfunction of CD8^+^ CTLs with high levels of CTLA-4 and PD-1 expression rather than CD4^+^ CTLs. (A and B) allogeneic mice were immunized with LICM-treated MSCs, splenocytes were isolated and labeled with fluorochrome-conjugated anti-CD4, CD8, Fas-L, Perforin, GzmB, CTLA-4, PD-1 mAbs, CTLA-4 and PD-1 expression of CD4^+^ CTLs (CD4^+^Fas-L^+^ CTL, CD4^+^Perforin^+^ CTL) and CD8^+^ CTLs (CD8^+^GzmB^+^ CTL, CD8^+^Perforin^+^ CTL) was analyzed by flow cytometry (n = 3). The results are representative of three independent experiments as mean ± SD. ^**^p < 0.01. (C and D) after immunization, isolated splenocytes were co-cultured with LICM-treated MSCs and labeled with Annexin V, 7-AAD and anti-CD4, CD8, Fas-L, Perforin, GzmB mAbs, survival of CD4^+^ CTLs and CD8^+^ CTLs was analyzed by flow cytometry (n = 3). The results are representative of three independent experiments as mean ± SD. ^*^p < 0.05, ^**^p < 0.01. (E) MSCs were treated with LICM and labeled with anti-CD80, CD86, PD-L1, Fas mAbs, expression of CD80, CD86, PD-L1 and Fas on MSCs was analyzed by flow cytometry (n = 3). The results are representative of three independent experiments as mean ± SD. ns = non-significant, ^*^p < 0.05, ^**^p < 0.01.

MHC-I and MHC-II have been proven as the critical molecules that provoke allograft rejection over past decades [50, 51]. Our results indicated that MHC-II knockout dramatically rescues the rejection of allografted MSCs, whereas the function of MHC-I is limited, despite its upregulation under liver-injury stimulation (Supplementary Fig. 1C). Given that CD8^+^ T cells express remarkably higher levels of inhibitory molecules in tumor models [52]. Blockade of CTLA-4 and PD-1 leads to reversal of CD8^+^ T cell dysfunction and subsequently tumor rejection [53]. Therefore, to analyze the role of inhibitory molecules in restricting the function of MHC-I rather than MHC-II in MSC allograft rejection, we evaluated the expression levels of CTLA-4 and PD-1 on CD8^+^ CTLs and CD4^+^ CTLs. Flow cytometric analysis demonstrated the remarkably higher expression levels of CTLA-4 and PD-1 on CD8^+^ CTLs than on CD4^+^ CTLs (Fig. 7A and B). Furthermore, co-culture with LICM-treated MSCs led to dramatically higher death rate of CD8^+^ CTLs than of CD4^+^ CTLs (Fig. 7C and D). In addition, ligands of CTLA-4 and PD-1 expressed on MSCs were measured by flow cytometry. As expected, LICM-treatment significantly upregulated CD80, PD-L1 and Fas expression of MSCs (Fig. 7E). These results suggested that MSCs upregulated CD80 and PD-L1 expression under liver injury condition. Thus, significant apoptosis of CD8^+^ CTLs with high levels of CTLA-4 and PD-1 expression rather than CD4^+^ CTLs was induced, thereby implying the mechanism of impaired function of MHC-I in MSC allograft rejection.

### Improvement of therapeutic effects of MHC-II^-/-^ allo-MSCs on liver injury

**Figure 8.**
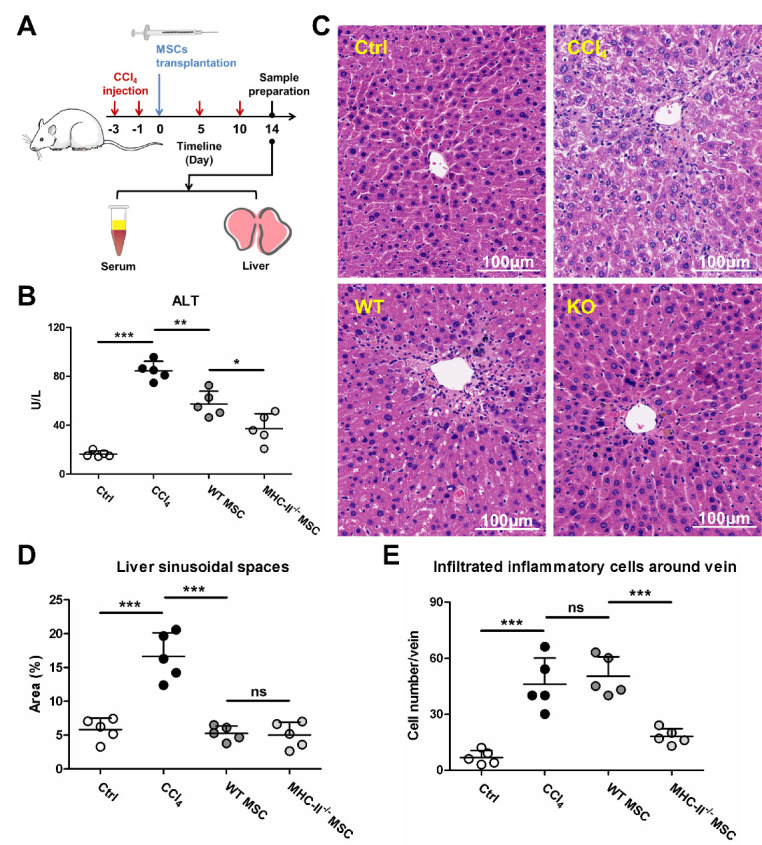
MHC-II knockout of allografted MSCs benefits hepatic injury repair. (A) schematic for preparation of serum and liver samples from mice with CCl_4_-induced hepatic injury after intravenous transplantation of allo-MSCs. (B) liver-injured mice received transplantation of allogeneic WT MSCs and MHC-II^-/-^ MSCs respectively, and liver function was evaluated by serum ALT level (n = 5). Ctrl, healthy mice. CCl_4_, CCl_4_-induced liver-injured mice without MSC transplantation. The results are representative of at least three independent experiments as mean ± SD. ns = non-significant, ^*^p < 0.05, ^**^p < 0.01, ^***^p < 0.001. (C-E) liver tissues were sectioned for histological H&E staining (C), area of sinusoidal spaces (D) and cell number of infiltrated inflammatory cells (E) were quantified from random non-overlapping fields of each group (n = 5) by ImageJ software. The results are representative of at least three independent experiments as mean ± SD. ns = non-significant, ^***^p < 0.001.

**Figure 9.**
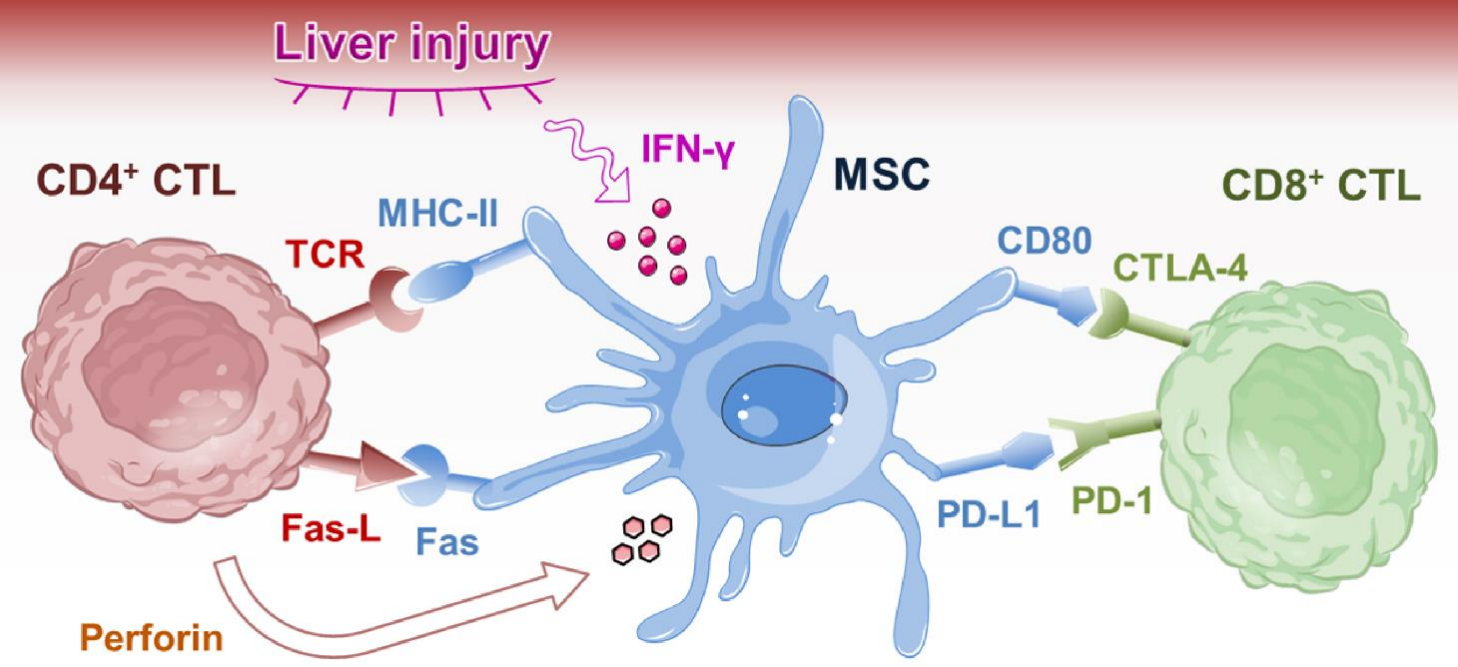
Model of CD4^+^ CTL-mediated immune rejection of allografted MSCs with upregulated immunogenicity under liver injury condition. Allografted MSCs upregulated MHC-II upon stimulation of IFN-γ in liver injury environment. MHC-II upregulation enhanced the immunogenicity of allo-MSCs, leading to the activation of alloreactive CD4^+^ CTLs and rejection of allo-MSCs. On the other hand, allo-MSCs upregulated CD80 and PD-L1 under liver injury condition while CD8^+^ CTLs highly expressed CTLA-4 and PD-1, thereby inducing immune tolerance of CD8^+^ CTLs to allo-MSCs.

As evidenced above, allo-MSCs induced CD4^+^ CTL-mediated rejection by upregulating MHC-II and suppressed CD8^+^ CTL function by upregulating PD-L1 under liver injury condition; MHC-II knockout promoted survival of allografted MSCs. Therefore, we tested whether the MHC-II-knockout (MHC-II^-/-^) allo-MSCs can improve their therapeutic effects on liver injury. For this purpose, WT C57BL/6 mice-derived MSCs (WT allo-MSCs) and MHC-II^-/-^ C57BL/6 mice-derived MSCs (MHC-II^-/-^ allo-MSCs) were intravenously transplanted into BALB/c mice with CCl_4_-induced hepatic injury, respectively. After transplantation for 14 days, serum and liver samples were collected for the tests (Fig. 8A). As a result, transplantation of WT allo-MSCs exhibited remarkably low level of serum alanine aminotransferase (ALT) in contrast to non-transplanted group. However, transplantation of MHC-II^-/-^ allo-MSCs significantly decreased the ALT level compared with WT allo-MSCs (Fig. 8B). In addition, transplantation of WT allo-MSCs significantly attenuated CCl_4_-induced hepatocyte necrosis and empty sinusoidal spaces. However, the disarrangement of hepatic parenchyma was not ameliorated, and was accompanied by infiltration of inflammatory cells around the vein. In contrast, transplantation of MHC-II^-/-^ allo-MSCs showed normal hepatic architecture and remarkably attenuated infiltration of inflammatory cells (Fig. 8C-E). These results demonstrated that MHC-II knockout of allografted MSCs improves their therapeutic effects on liver injury. In summary, we discovered that CD4^+^ CTLs mediate immune rejection of allografted MSCs with MHC-II upregulation under liver injury condition, while MHC-II knockout of MSCs prevents allograft rejection and benefits hepatic injury repair (Fig. 9). These findings revealed a previously unrecognized mechanism of allo-MSCs rejection after implantation into injured liver, which provided strategies for improving therapeutic effects of allo-MSCs on liver injury.

## DISCUSSION

In recent studies, administration of MSCs holds great promise as a cytotherapy for liver diseases [41]. Under most acute clinical conditions, allo-MSCs from healthy donors would be the most suitable option because they can be selected according to the therapeutic performance and prepared in advance [14, 15, 54]. Numbers of *in vitro* experiments revealed the low immunogenicity and potent immunomodulatory capacity of MSCs; thus, they are plausibly immunoprivileged [22, 24, 55, 56]. Nevertheless, accumulating *in vivo* results indicated that allo-MSCs elicit immune response and cause rejection after transplantation, thereby limiting their long-term benefits [25, 28, 57]. Our data showed that allo-MSCs upregulated MHC-II in CCl_4_-induced acutely injured mouse liver after intravenous transplantation. We simulated the acute liver injury condition by using LICM *in vitro* and discovered that MSCs upregulated MHC-II expression under treatment with low concentration levels of LICM, while downregulated MHC-II expression under high concentrations of LICM. Furthermore, we identified IFN-γ as the key factor of LICM in upregulating MHC-II of MSCs and found that MHC-II expression was induced by low levels of IFN-γ, but inhibited by elevated levels of IFN-γ, this outcome was consistent with the pattern of LICM-induced MHC-II expression. Given that IFN-γ is one of the most important pro-inflammatory cytokines in injured liver, these findings may explain the reason why MSCs minimally expressed MHC-II at the early stage of liver injury (7 days after transplantation), while remarkably expressed MHC-II at the later stage of injury (14 days after transplantation). In this case, CCl_4_-induced acutely hepatic injury caused the burst of inflammatory cytokines with abundant IFN-γ. During the first 7 days after MSC transplantation, the high level of IFN-γ in the inflamed liver inhibited the MHC-II expression of MSCs. After transplantation for 14 days, MSCs alleviated liver inflammation accompanied with a significant decrease in IFN-γ [58, 59]. Consequently, the low level of IFN-γ allowed a rapid MHC-II expression in MSCs. It is worth mentioning that the inhibitory effect of high IFN-γ level on MHC-II expression could be partly explained by the cytoplasmic retention of CIITA, a master transcription activator of MHC-II, as reported previously [60]. Finally, we found that MHC-II upregulation enhanced the immunogenicity and resulted in rejection of allo-MSCs, while MHC-II knockout failed to stimulate lymphocyte proliferation and cytotoxicity, thereby remarkably rescuing the loss of allo-MSCs after transplantation. Thus, infused allo-MSCs exhibited enhanced immunogenicity and suffered immune rejection in certain inflammatory microenvironment, rather than displayed an immunoprivileged behavior all the time.

CD4^+^ T cells are traditionally regarded as helpers that regulate various immune cells. However, a novel role of CD4^+^ T cells as effectors has emerged. CD4^+^ cytotoxic T lymphocytes (CTLs) are a subpopulation of CD4^+^ T cells with direct cytolytic activity, which have been reported to function in anti-viruses and anti-tumors [61, 62]. Moreover, CD4^+^ CTLs contribute to allograft rejection with their strong allospecific cytotoxicity [63, 64]. Here, we showed that allografted MSCs caused proliferation and infiltration of CD4^+^ CTLs in a MHC-II-dependent manner, which largely contributed to the allograft rejection of MSCs through direct cytotoxic activity. This notion was supported by the observation that the knockout of MHC-II on allo-MSCs sufficiently inhibited the allo-MSC-elicited proliferation and cytotoxicity of CD4^+^ CTLs, which eventually rescued allo-MSCs from rejection. In addition, adoptive transfer of CD4^+^ CTLs significantly decreased the survival of allo-MSCs implanted in nude mice; however, adoptive transfer of CD45^+^CD4^-^ leukocytes that contain various immune cells except CD4^+^ T cells did not have such an impact. Meantime, no remarkable infiltration of activated CD8^+^CD107a^+^ T cells and CD19^+^CD86^+^ B cells was detected in allo-MSC-implanted mice along with the adoptive transfer of CD45^+^CD4^-^ leukocytes. These findings indicate that the alloreactive CD4^+^ CTLs act as a key effector population for allograft rejection of MSCs, which may directly reject the allo-MSCs without intervention of other immune cells. Notably, the immunogenic effect of MHC-I was minimized, as shown by the anergy of CD8^+^ CTLs during the allo-MSC rejection. Hence, we evaluated the possible differential regulatory role of immune-checkpoint inhibitors in the activation of CD8^+^ and CD4^+^ CTLs under liver injury condition. As expected, CD8^+^ CTLs rather than CD4^+^ CTLs expressed high levels of CTLA-4 and PD-1 co-inhibitory receptors. Accordingly, allo-MSCs upregulated CD80 and PD-L1, thereby inducing immune tolerance of CD8^+^ CTLs to allo-MSC, while CD4^+^ CTLs remained cytotoxic to allo-MSCs. As a consequence, transplantation of MHC-II-deficient (MHC-II^-/-^) allo-MSCs significantly improved their therapeutic outcomes in treating liver injury by preventing attack from CD4^+^ CTLs. Thus, the use of genetically engineered MHC-II^-/-^ allo-MSCs or intervention of MHC-II-targeting CD4^+^ CTLs is an attractive strategy for preventing allo-MSCs from allograft rejection, thereby greatly benefiting the long-term application of allo-MSCs in liver injury repair. On the other hand, PD-L1 was found to be remarkably induced on allo-MSCs in liver injury condition. Thus, the allo-MSCs with high PD-L1 expression level can provide strong coinhibitory signals for inflammatory cells with high expression level of PD-1. This observation may represent an important mechanism underlying MSC-mediated immunosuppressive regulation for hepatic inflammation. Moreover, IFN-γ treatment can induce PD-L1 expression, which had been documented previously [65]. Hence, INF-γ pre-treatment will greatly improve the anti-inflammatory activity of allo-MSCs by enhancing their expression of PD-L1. In this case, MHC-II^-/-^ allo-MSCs are preferred, considering that INF-γ induced the expression of both PD-L1 and MHC-II, among which the upregulation of MHC-II is absolutely undesirable. In summary, our study revealed the contribution of CD4^+^ CTLs to the allograft rejection of MSCs with MHC-II upregulation under liver injury condition. This finding provided strategies for improving clinical performance of allo-MSCs in benefiting hepatic injury repair.

## Supplementary Materials

The Supplementary data can be found online at: www.aginganddisease.org/EN/10.14336/AD.2022.0313.
