# Supplementary material for: CD4+ CTLs Act as a Key Effector Population for Allograft Rejection of MSCs in a Donor MHC-II Dependent Manner in Injured Liver
Source: Aging Dis. 2022 Dec 1;13(6):1919–38. doi: 10.14336/AD.2022.0314 (PMC9662282; doi:10.14336/AD.2022.0314)
Supplement: Supplementary file 1 — The Supplementary data can be found online at: www.aginganddisease.org/EN/10.14336/AD.2022.0313. [file AD-13-6-1919-s.pdf]

SUPPLEMENTARY DATA

$CD4^+$ CTLs Act as a Key Effector Population for Allograft Rejection of MSCs in a Donor MHC-II Dependent Manner in Injured Liver

Shuang Shen[1], Yuanhui Li[1], Mengting Jin[1], Dongdong Fan[1], Ruolang Pan[4], Aifu Lin[1], Ye Chen[3*], Lixin Xiang[1*], Robert Chunhua Zhao[2*], Jianzhong Shao[1*]

© 2022. Shen S et al. Published online at http://www.aginganddisease.org/EN/10.14336/AD.2021.0314

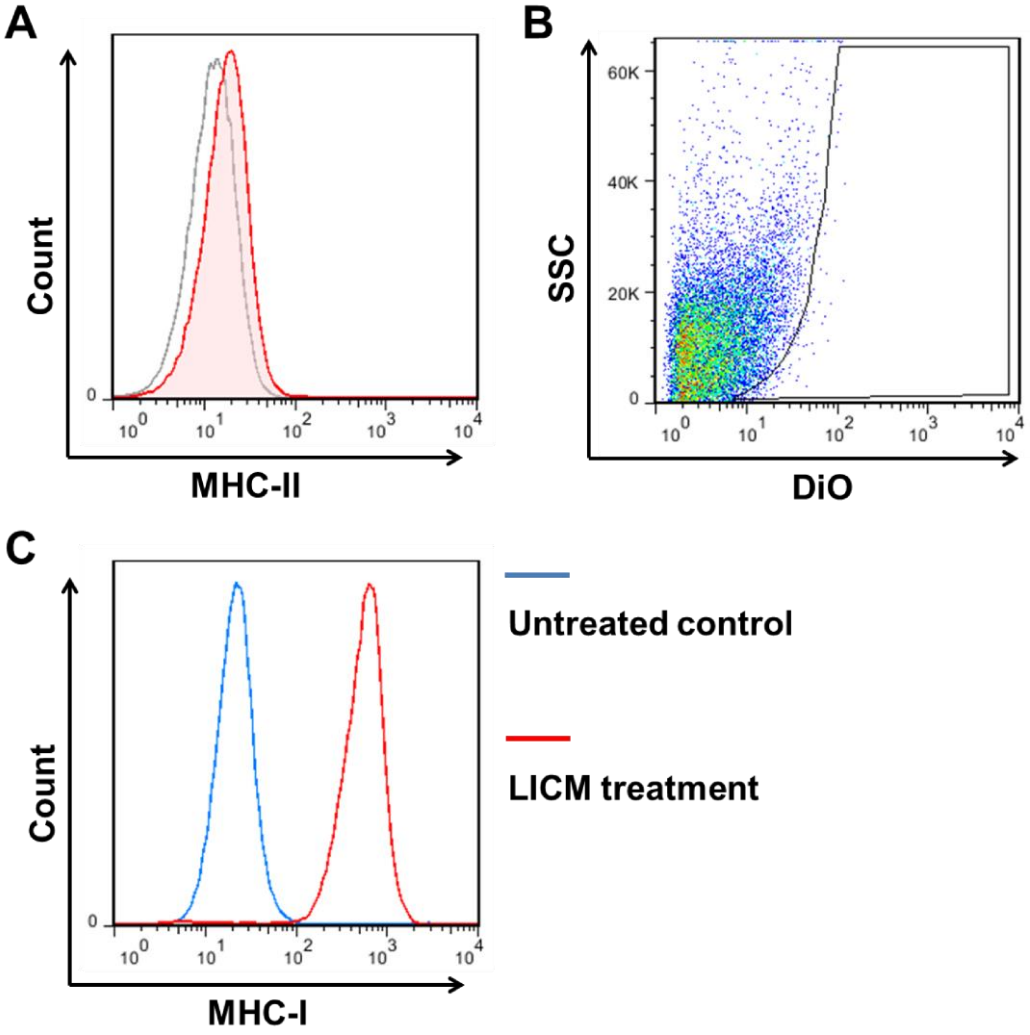

Supplementary Figure 1. Examination of MHC-II, MHC-I expression on MSCs and implantation of MSCs in non-injured liver. ***A,*** flow cytometric analysis for MHC-II expression on MSCs before transplantation. ***B,*** flow cytometric analysis for implantation of MSCs in non-injured liver. MSCs (5×10^5) were labeled with DiO and transplanted into mice with non-injured liver through tail vein. After transplantation for 14 days, liver was harvested, and hepatic non-parenchymal cells were isolated for flow cytometric analysis. ***C,*** flow cytometric analysis for MHC-I expression on MSCs after LICM treatment for 3 days.

© 2022. Shen S et al. Published online at http://www.aginganddisease.org/EN/10.14336/AD.2021.0314

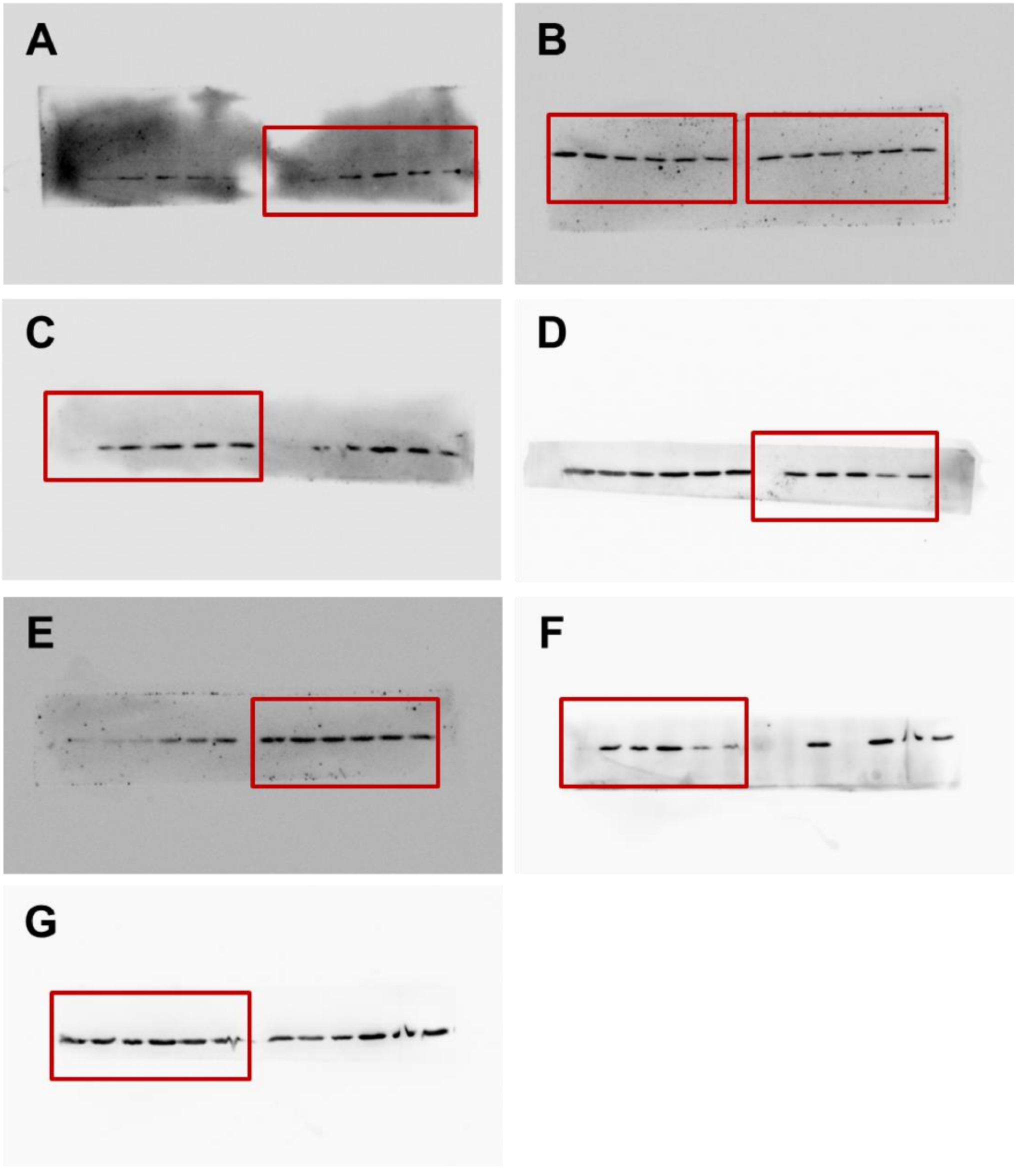

Supplementary Figure 2. Uncropped original images for Western blot analysis. ***A,*** immunoblot bands of MHC-II in Figure 3C (right 6 lanes in red frame). ***B,*** immunoblot bands of GAPDH in Figure 3C (right 6 lanes in red frame) and Figure 3H (left 6 lanes in red frame). ***C,*** immunoblot bands of MHC-II in Figure 3H (left 6 lanes in red frame). ***D,*** immunoblot bands of MHC-II in Figure 4C (right 6 lanes in red frame). ***E,*** immunoblot bands of GAPDH in Figure 4C (right 6 lanes in red frame). ***F,*** immunoblot bands of MHC-II in Figure 4H (left 6 lanes in red frame). ***G,*** immunoblot bands of GAPDH in Figure 4H (left 6 lanes in red frame).

© 2022. Shen S et al. Published online at http://www.aginganddisease.org/EN/10.14336/AD.2021.0314

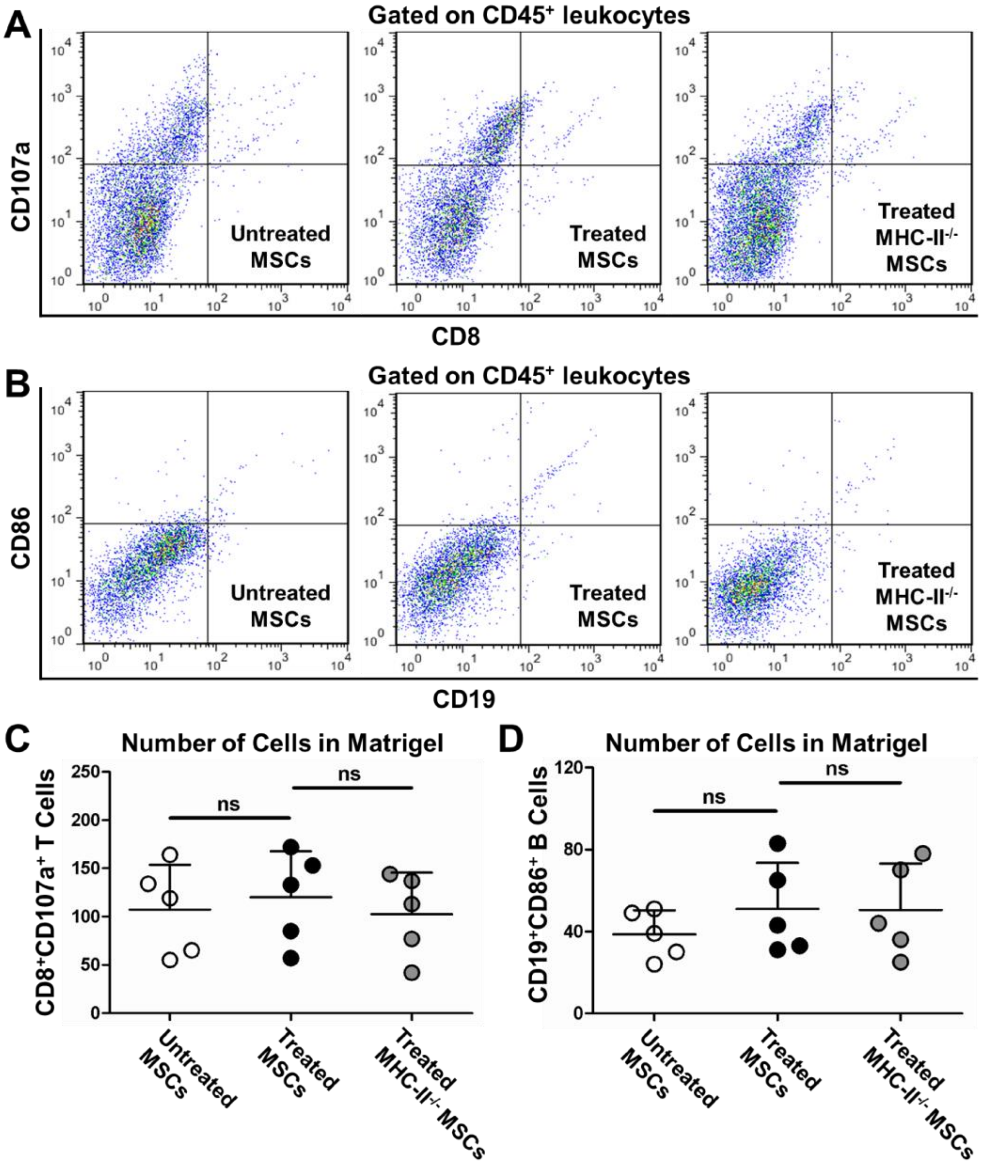

Supplementary Figure 3. Examination on the infiltration of activated CD8$^+$ T cells and B cells in Matrigel embedded with allo-MSCs. ***A*** and ***C***, flow cytometric analysis for cell number of infiltrated CD8$^+$CD107a$^+$ T cells (n = 5). ***B*** and ***D***, flow cytometric analysis for cell number of infiltrated CD19$^+$CD86$^+$ B cells (n = 5). Untreated MSCs, LICM-treated MSCs and LICM-treated MHC-II$^{-/-}$ MSCs were mixed with Matrigel respectively and injected subcutaneously in allogeneic mice. Matrigel was retrieved 7 days after injection; infiltrated cells were isolated and labeled with anti-CD45, CD8, CD107a mAbs or anti-CD45, CD19, CD86 mAbs. The results are representative of at least three independent experiments as mean ± SD. ns = non-significant.

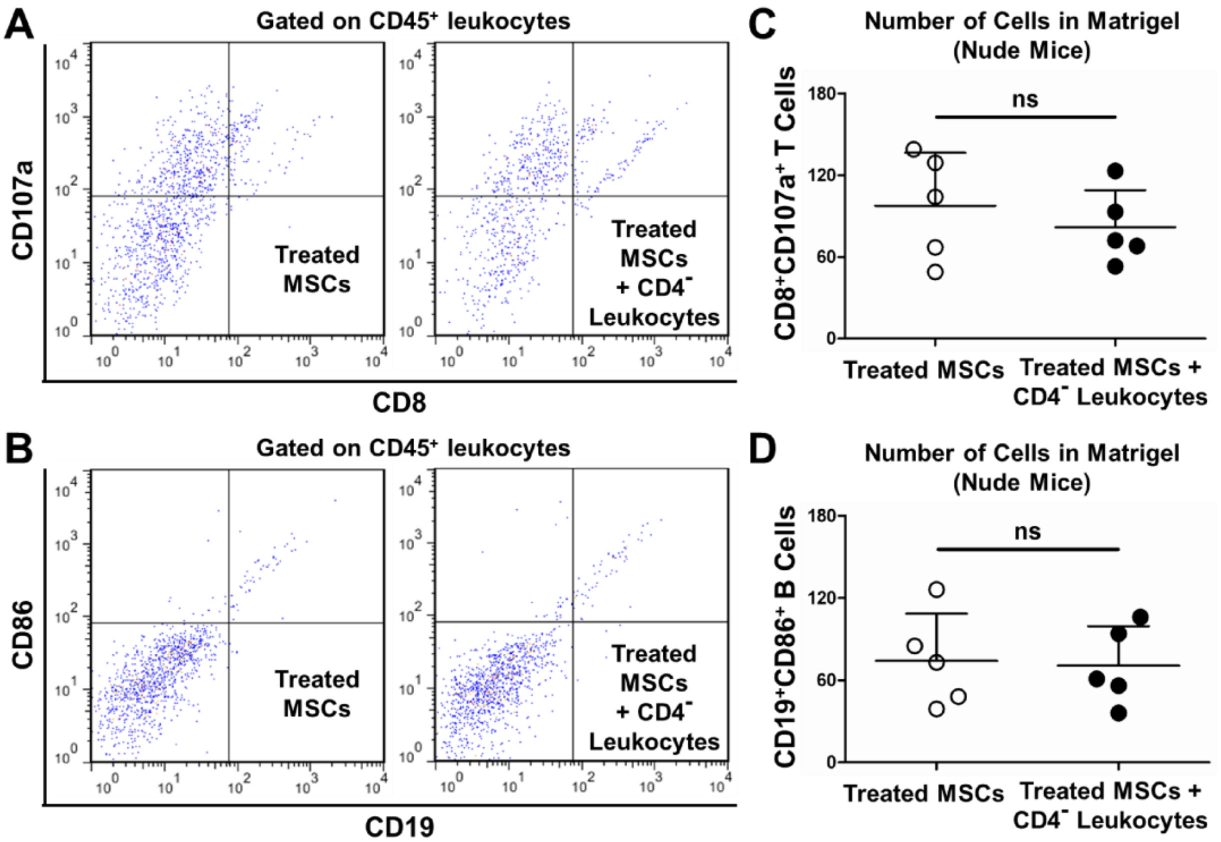

Supplementary Figure 4. Examination on the infiltration of activated CD8$^+$ T cells and B cells in Matrigel embedded with allo-MSCs in nude mice. ***A*** and ***C***, flow cytometric analysis for cell number of infiltrated CD8$^+$CD107a$^+$ T cells (n = 5). ***B*** and ***D***, flow cytometric analysis for cell number of infiltrated CD19$^+$CD86$^+$ B cells (n = 5). MSCs were treated with LICM, mixed with Matrigel and injected subcutaneously in allogeneic nude mice with/without adoptive transfer of CD45$^+$CD4$^-$ leukocytes. Matrigel was retrieved 7 days after injection; infiltrated cells were isolated and labeled with anti-CD45, CD8, CD107a mAbs or anti-CD45, CD19, CD86 mAbs. The results are representative of at least three independent experiments as mean ± SD. ns = non-significant.

© 2022. Shen S et al. Published online at http://www.aginganddisease.org/EN/10.14336/AD.2021.0314
